# Health care and social media: What patients really understand

**DOI:** 10.12688/f1000research.10637.1

**Published:** 2017-02-08

**Authors:** Kyle Hoedebecke, Lindsey Beaman, Joy Mugambi, Sanam Shah, Marwa Mohasseb, Cheyanne Vetter, Kim Yu, Irini Gergianaki, Emily Couvillon

**Affiliations:** 1 WONCA Polaris, Seoul, Korea, South; 2 Leidos Biomedical Research, Inc., Frederick, MD, USA; 3 Afriwon Renaissance Movement, Naivasha, Kenya; 4 The Spice Route Movement, Karachi, Pakistan; 5 Al Razi Movement, Cairo, Egypt; 6 WONCA Polaris, Saskatchewan, Canada; 7 Covenant Healthcare, Frankenmuth, MI, USA; 8 University of Crete, Crete, Greece; 9 Texas Medical Center Library, Houston, TX, USA

**Keywords:** Social Media, Twitter, Web 2.0, health literacy, patient comprehension

## Abstract

*Background*: Low health literacy is associated with decreased patient compliance and worse outcomes - with clinicians increasingly relying on printed materials to lower such risks. Yet, many of these documents exceed recommended comprehension levels. Furthermore, patients look increasingly to social media (SoMe) to answer healthcare questions. The character limits built into Twitter encourage users to publish small quantities of text, which are more accessible to patients with low health literacy. The present authors hypothesize that SoMe posts are written at lower grade levels than traditional medical sources, improving patient health literacy. *Methods:* The data sample consisted of the first 100 original tweets from three trending medical hashtags, leading to a total of 300 tweets. The Flesch-Kincaid Readability Formula (FKRF) was used to derive grade level of the tweets. Data was analyzed via descriptive and inferential statistics. *Results:* The readability scores for the data sample had a mean grade level of 9.45. A notable 47.6% of tweets were above ninth grade reading level. An independent-sample t-test comparing FKRF mean scores of different hashtags found differences between the means of the following: #hearthealth versus #diabetes (t = 3.15, p = 0.002); #hearthealth versus #migraine (t = 0.09, p = 0.9); and #diabetes versus #migraine (t = 3.4, p = 0.001). *Conclusions:* Tweets from this data sample were written at a mean grade level of 9.45, signifying a level between the ninth and tenth grades. This is higher than desired, yet still better than traditional sources, which have been previously analyzed. Ultimately, those responsible for health care SoMe posts must continue to improve efforts to reach the recommended reading level (between the sixth and eighth grade), so as to ensure optimal comprehension of patients.

## Introduction

Health literacy - defined as the degree to which an individual has the capacity to obtain, communicate, process, and understand basic health information and services to make appropriate health decisions - is considered to be the single best predictor of an individual’s health status ( http://www.cdc.gov/healthliteracy/learn/) ^1^. Low health literacy correlates with decreased patient compliance and poorer outcomes, leading to an increase in clinician reliance on printed materials to mitigate such risks ^2^. Yet, a recent study identified that many of these materials exceed the recommended sixth to eighth grade reading level of the American Medical Association (AMA), National Institute of Health (NIH) and Center for Disease Control and Prevention (CDC) ( http://www.nlm.nih.gov/medlineplus/etr.html; http://www.cdc.gov/DHDSP/cdcynergy_training/Content/activeinformation/resources/simpput.pdf) ^3, 4^. As medical vocabulary becomes more integrated into social media (SoMe), the healthcare community must remember to employ comprehensible language when engaging audiences through platforms such as Facebook, Twitter, and LinkedIn.

Generally, patients are increasingly relying on SoMe as a primary avenue for answering healthcare questions ^5, 6^. For example, this may be due to the character limits built into Twitter that encourage users to publish small chunks of text, which are increasingly accessible to patients with low health literacy ^7^. As health literacy directly impacts patient outcomes, it remains imperative for healthcare providers to intentionally tailor their writing level of SoMe posts to enhance patient-centred communication and comprehension.

The present authors hypothesized that SoMe posts on the Twitter platform are written at a lower grade level than traditional medical sources, allowing for better patient health literacy.

## Methods

The data sample consisted of the first 100 original tweets in 2016 via the pay-to-access Symplur Signal analytics tools ( http://www.symplur.com/signals/) from each of the March 2016 top trending hashtags: #hearthealth, #diabetes and #migraines, leading to a total of 300 tweets being analyzed. Trending hashtags related to primary care were selected, as these tweets would have the greatest impact and overall reach worldwide. Exclusion criteria included non-English or non-medical tweets, as well as those that encompassed links with non-medical webpages or product advertisements.

The Flesch-Kincaid Readability Formula (FKRF) is a validated tool to assess the grade level of written material and is calculated with the following formula: 206.835 - 1.015 (total words/total sentences) - 84.6 (total syllables/total words). The FKRF Grade Level Scores can be interpreted as shown in Table 1 between the fifth grade to graduate levels ^8^. Each tweet was evaluated via FKRF to derive grade level. SPSS (version 21.0 for Mac; http://www.ibm.com/analytics/us/en/technology/spss/) was used for data analysis, and data was analyzed using descriptive and inferential statistics. Descriptive statistics included the mean with 95% confidence interval, median, range and standard deviation of FKRF scores. All *p* values were derived from two-sided *t*-tests. The project was approved by Stanford’s IRB and Medical Ethics Team, as a part of the 2016 Stanford MedX/Symplur Social Media Competition.

**Table 1.** Reading difficulty rating of Flesch-Kincaid Readability Formula grade level scores ^8^.

Flesch-Kincaid grade level score	Reading difficulty rating
5th	Very easy to read
6th	Easy to read
7th	Fairly easy to read
8th and 9th	Plain English/ standard
10th to 12th	Fairly difficult to read
College/13th–16th	Difficult to read
College graduate and beyond	Very difficult to read

## Results

The readability scores for the 300 total tweets evaluated are presented in Table 2. The mean FKRF grade level was 9.45, signifying a level between the ninth and tenth grades. A notable 47.6% of tweets were above the ninth grade reading level ( Table 2). There was a wide range of FKRF scores, as shown in Table 3, varying from elementary to postgraduate levels.

An independent-sample t *-*test ** comparing the FKRF mean scores of different hashtags found differences between the means of groups as follows: #hearthealth versus #diabetes ( *t* = 3.15, *p* = 0.002); #hearthealth versus #migraine ( *t* = 0.09, *p* = 0.9); and #diabetes versus #migraine ( *t* = 3.4, *p* = 0.001). Therefore, there was a significant difference between the means of two groups: #hearthealth versus #diabetes, and #diabetes versus #migraine. Although it is unclear why the differences exist, this identifies that the grade level comprehension varies significantly when dealing with tweets surrounding differing health issues. One such explanation could be the differing characteristics of the tweet author and their health care experience. Additionally, the differing incidences of migraines and heart disease may affect the availability of reading materials as well as the grade level at which each is written.

**Table 2.** Flesch-Kincaid Readability Formula (FKRF) grade level scores for the total sample.

Total sample (n=300)	FKRF grade level
Mean	9.45
Median	9.05
Standard deviation	4.95
Range	1.2 – 28.4

**Table 3.** Tweet material grade level summary by Flesch-Kincaid Readability Formula (FKRF) (n=300).

Grade level	FKRF, n (%)
1st – 3rd	32 (10.7)
4th – 6th	59 (19.7)
7th – 9th	66 (22.0)
10th – 12th	70 (23.3)
>12th	73 (24.3)

The 300 tweets analysed by the present study divided by #migraine, #hearthealth and #diabetes Click here for additional data file. Copyright: © 2017 Hoedebecke K et al. 2017 Data associated with the article are available under the terms of the Creative Commons Zero "No rights reserved" data waiver (CC0 1.0 Public domain dedication).

Raw data for SPSS Click here for additional data file. Copyright: © 2017 Hoedebecke K et al. 2017 Data associated with the article are available under the terms of the Creative Commons Zero "No rights reserved" data waiver (CC0 1.0 Public domain dedication).

## Discussion

SoMe - especially Twitter - is a cost-effective, interactive communication tool with increasing applicability within the medical sector ^9^. Although limited health literacy of the audience poses a real threat in disseminating health messages, few studies have examined the readability of Twitter healthcare posts for the general public. In the present study, the authors found that a Twitter sample (n=300) was written at a mean of FKRF grade level 9.45, signifying a level between the ninth and tenth grade ( Table 1). This outcome proves much closer to the NIH readability goal, as compared to previous studies that found patient medical consent forms to be written between the eleventh to thirteenth grade levels (three to five grades higher than the current NIH recommendation), and on major associations’ websites and educational materials, which were written above the recommended reading level ( http://www.nlm.nih.gov/medlineplus/etr.html).

 One potential reason for this outcome lies in Twitter’s character limit itself, which permits only 140 characters to be written. Undoubtedly, this may prove a double-edged sword as this limitation creates a more manageable length, but also forces the composer to employ more concise terminology carrying a more complex readability factor. Given the increasing number of Twitter users, readability should be further evaluated towards meaningful health messaging, diminishing disparities in comprehension and ameliorating patient difficulties to understand and follow instructions and recommendations.

This study has some limitations, including the relatively small sample, the use of a single readability scale and a single SoMe platform. On the other hand, there are major strengths, as our study provides an updated focus of readability of web 2.0 communication tools. The findings highlight the possibility that Twitter can be a way of reaching the readability guidelines, as compared to written educational materials or online materials on websites. Twitter was used as a model, but more platforms on SoMe should be evaluated, so that guidelines could be shaped to recognize the unmet needs of health communication in a modern era. Ultimately, those responsible for health care SoMe and other relevant platforms posts must continue to improve efforts to reach the recommended reading level, so as to ensure optimal comprehension and enhance the capacity of patients and doctors to mutually interact.

## Conclusions

The sample studied identifies that health care SoMe posts allow for better patient health literacy than traditional medical sources. Health care advocates must remain vigilant, so that posts improve upon current readability levels. Lastly, respectable medical sources should consider additional use of SoMe avenues to dispense more comprehensible health care information to a wider patient audience.

## Data availability

The data referenced by this article are under copyright with the following copyright statement: Copyright: © 2017 Hoedebecke K et al.

Data associated with the article are available under the terms of the Creative Commons Zero "No rights reserved" data waiver (CC0 1.0 Public domain dedication). 

Dataset 1: The 300 tweets analysed by the present study divided by #migraine, #hearthealth and #diabetes. doi, 10.5256/f1000research.10637.d150437 ^10^

Dataset 2: Raw data for SPSS. doi, 10.5256/f1000research.10637.d150438 ^11^
