# Supplementary material for: Health care and social media: What patients really understand
Source: F1000Res. 2017 Feb 8;6:118. [Version 1] doi: 10.12688/f1000research.10637.1 (PMC5381615; doi:10.12688/f1000research.10637.1)
Supplement: Peer review history

# Peer review: Referee response for version 1

**DOI:** 10.5256/f1000research.11463.r20791

**Published:** 2017-03-30

**Authors:** Shabir A. H. Moosa

**Affiliations:** 1 Department of Family Medicine, University of the Witwatersrand, Johannesburg, Johannesburg, South Africa

Congrats to this global set of young authors from the Wonca circle! Really good application of research method to an important issues.

 There are some suggestions that would help the authors.

 1. The authors say “ present authors hypothesize that SoMe posts are written at lower grade levels than traditional medical sources, improving patient health literacy”. The research method, results, and discussion do not reflect that with no clarity as to what ‘traditional medical sources” are compared with. Reference to previous studies under discussion is referenced to the MedlinePlus webpage on “How to write…” with no clear evidence of ‘traditional medical sources’ readability scores with the FKRF. Printed material and websites would have been a useful comparator vs. consent forms. The comparison of different hashtags and statistical tests seem irrelevant/tangential to the hypothesis or enquiry.

 2. The choice of Twitter and assumptions about it ie. small chunks of text making it more accessible are contradicted later when the Twitter limitations of complex readability factor are later related due to small chunks. It would have been useful to relate to global use of SoMe platforms, by population and possibly patients.

 3. The method of using the FKRF is not clearly articulated and seems difficult to relate to Table 2 readability score.

 4. The conclusion "The sample studied identifies that health care SoMe posts allow for better patient health literacy than traditional medical sources.” seems largely unsupported.

 Keep it up.

I have read this submission. I believe that I have an appropriate level of expertise to confirm that it is of an acceptable scientific standard.

# Peer review: Referee response for version 1

**DOI:** 10.5256/f1000research.11463.r20061

**Published:** 2017-03-07

**Authors:** Shailendra Prasad

**Affiliations:** 1 Dept of Family Medicine and Community Health, University of Minnesota, Minneapolis, MN, USA

I compliment the authors on this study. It is fascinating to see the group from various parts of the world collaborate on this project. 

 - The project/manuscript is a much-needed one with the rapid growth of social media in society. There are indications that access to social media platforms have increased dramatically in the last decade and further democratizes information sharing.

 - The authors use a novel approach to look at the content of various social media posts in Twitter regarding health and determine that the readability of the posts are much better than conventional health education methods.

 - The article is well written and easily readable.

 I have a couple of minor corrections to recommend.

 1) Highlight that this analysis is particular to Twitter. The authors have done this, but I would recommend reinforcing this as the title indicates Social Media ( which has different platforms). 

 2) The issue then is possibly of brevity rather than the platform. I would recommend that the authors highlight this in the discussion too.

 3) I recommend that the authors highlight the other issues in understanding health-related information, including the assimilation of the information and behavior change that needs to follow and that further studies should look at this.

 Again, my compliments to the authors.

I have read this submission. I believe that I have an appropriate level of expertise to confirm that it is of an acceptable scientific standard.
